# Multifunctional Roles and Ecological Implications of Nano-Enabled Technologies in *Oryza sativa* Production Systems: A Comprehensive Review

**DOI:** 10.3390/plants14040528

**Published:** 2025-02-09

**Authors:** Wei Zhao, Ting Wang, He Dong, Wanru Zhao, Kai Song, Nina Zhu

**Affiliations:** 1 School of Life Science, Changchun Normal University, Changchun 130032, China; weiweizcn@163.com (W.Z.); mdonghe@163.com (H.D.); zhaowanrumei@163.com (W.Z.); 2 School of Agricultural Engineering, Shanxi Agricultural University, Jinzhong 030810, China; 17735460379@163.com; 3 Institute of Innovation Science and Technology, Changchun Normal University, Changchun 130032, China

**Keywords:** micro–nanomaterials, rice cultivation, ecological safety, sustainable agriculture, pollution

## Abstract

Micro–nanomaterials have garnered significant attention in rice (*Oryza sativa* L.) cultivation due to their unique physicochemical properties. This study reviews the multifunctional applications of micro–nanomaterials in enhancing rice resilience, promoting nutrient uptake, improving photosynthetic efficiency, and increasing the utilization rates of fertilizers and pesticides. Using keyword and clustering analyses, this review identifies key research hotspots and emerging trends in the field, including heavy metal stress, nanoplastic pollution, and biochar applications. While early studies predominantly focused on the synthesis and characterization of these materials, recent research has shifted towards evaluating their comprehensive ecological impacts on rice production systems. Despite the promising potential of micro–nanomaterials in improving rice yield and quality while supporting sustainable agriculture, concerns about their long-term accumulation in ecosystems and potential toxicity remain unresolved. Future research should prioritize the development of cost-effective, efficient, and environmentally friendly micro–nanomaterials and establish standardized frameworks for ecological risk assessments to facilitate their large-scale agricultural application. This study provides theoretical insights and practical references for advancing micro–nanotechnology in global food security and sustainable agriculture.

## 1. Introduction

Microscale and nanoscale materials, due to their unique physicochemical properties—including small size, high surface area, and exceptional catalytic performance—have demonstrated significant potential for applications in agriculture. These materials can regulate the release rate of fertilizers to improve nutrient utilization efficiency, enhance plant stress tolerance, and play a pivotal role in the remediation of contaminated soils [1,2,3]. Compared with traditional mineral amendments, micro–nanomaterials show unique advantages: (a) quantum size effect enhance targeted adsorption capacity; (b) surface modification for controlled release functionality; and (c) multilevel pore structure improve the permeability and water retention of soil [4]. These properties give them significant advantages in increasing fertilizer utilization, improving soil structure, and remediating contaminated soil. However, the high reactivity and quantum effects of micro–nanomaterials have raised environmental safety and health concerns while promoting sustainable agriculture. Improper disposal of production waste, material abrasion during use, and the absence of effective waste management mechanisms can result in the release of micro–nanoscale materials into air, water, and soil. Once introduced into the environment, these materials can persist, accumulate, and potentially cause long-term ecological impacts [5,6].

Nanoscale materials have demonstrated significant potential in rice (*Oryza sativa* L.) cultivation, offering benefits such as enhanced seed germination and accelerated seedling growth. For instance, carbon nanotubes, nano-iron oxide, and nanosilver have been shown to promote the production of plant growth hormones and enzymes, thereby stimulating root development and improving the uptake of nutrients and water [7,8,9,10,11,12]. Moreover, nanomaterials can influence plant physiological processes, strengthening their immunity and ability to withstand environmental stress [13]. Notably, nanosilicon and nanotitanium have been reported to improve stress tolerance by boosting photosynthetic efficiency and activating antioxidant enzymes [14]. However, despite these advantages, the use of nanomaterials is not without risks. Potential drawbacks include damage to plant cells and genetic material, which may hinder normal growth [15]. Additionally, the release of these materials into the environment could pose threats to the growth of rice and other crops [16].

At the microscale, microplastics (particles > 5 mm) have emerged as a pressing environmental pollutant. These particles primarily originate from the release of plastic granules or the fragmentation and aging of larger plastic debris, and they are now widely distributed in both aquatic and terrestrial ecosystems [17]. Research indicates that microplastics significantly influence the transport and transformation of pollutants in soils by altering soil physicochemical properties. For instance, the hydrophobicity of microplastics promotes the accumulation of organic pollutants on soil particle surfaces and, through weathering and aging, releases plastic additives that modify the adsorption behavior of pollutants like heavy metals [18,19]. Such changes pose direct threats to plant growth and may have cascading effects on agricultural ecosystems through pollutant accumulation.

Among staple crops, rice serves as an important model for studying the impacts of environmental pollution on agriculture due to its sensitivity to environmental changes. Studies have shown that micro–nanoscale material pollution can impair seed germination, photosynthesis, and nutrient absorption in rice by disrupting soil structure and root development, ultimately threatening crop yields. Additionally, these pollutants can alter soil microbial communities and soil physicochemical properties, exacerbating their negative effects. Understanding the mechanisms underlying the impacts of microplastic pollution on rice and exploring effective mitigation strategies are therefore of critical theoretical and practical significance.

To systematically assess the applications of micro–nanoscale materials in rice cultivation, this study reviews the research progress and trends in their interactions with rice. It identifies their potential mechanisms in mitigating environmental stresses and provides theoretical support for their sustainable use in agriculture. This work not only advances the practical application of micro–nanoscale materials in agriculture, but also contributes valuable insights for global food security and sustainable agricultural development.

## 2. Properties and Mechanisms of Micro–Nanomaterials in Agriculture

Micro–nanomaterials have shown significant potential in revolutionizing agriculture due to their unique physicochemical properties. These materials provide innovative solutions for key agricultural challenges, such as enhancing fertilizer efficiency, improving plant stress tolerance, promoting seed germination, and enabling precision farming through real-time monitoring. To limit the application of these materials in optimal quantities, several methodologies can be employed. First, controlled-release systems such as biodegradable dextrin-based microgel composites have been used to extend nutrient availability while minimizing nutrient loss [20]. Second, nano-sensors enable the real-time monitoring of soil, water, and plant health, ensuring the precise application of micro–nanomaterials [21]. Finally, nanoencapsulation technologies can be used to gradually release materials into the soil, ensuring that stimulants are applied without excess [22]. Table 1 summarizes the major functions of micro–nanomaterials in agriculture, highlighting their applications.

### 2.1. Classification and Properties of Micro–Nanomaterials

#### 2.1.1. Metallic Micro–Nanomaterials

Metallic nanomaterials, especially nanosilver, zinc oxide nanoparticles, and copper nanoparticles, represent one of the most common classes of micro–nanomaterials used in agriculture. These materials exhibit unique antimicrobial properties, which make them effective in controlling various plant diseases. For instance, nanosilver has been widely employed to suppress bacterial diseases in rice, demonstrating remarkable antimicrobial efficacy by inhibiting the growth and spread of pathogens [31]. Zinc oxide nanoparticles, on the other hand, enhance the growth potential of rice plants by promoting photosynthesis. Studies have shown that zinc oxide nanoparticles improve leaf light absorption and photosynthetic efficiency, thereby accelerating rice growth [32,33,34]. Additionally, microscale metal oxide particles, such as micronized iron oxide, have demonstrated excellent performance in soil improvement by immobilizing toxic ions such as cadmium and lead, reducing their harmful effects on crops [35,36]. However, excessive application of nanoparticles may inhibit plant growth and disrupt physiological functions. For example, excessive amounts of nano-copper oxide have been reported to decrease rice seed germination rates and cause oxidative stress in plants [37].

#### 2.1.2. Non-Metallic Micro–Nanomaterials

Non-metallic materials also exhibit significant application potential at micro–nanoscale dimensions. At the microscale, silica microspheres function as soil additives that enhance root nutrient absorption by improving soil water retention and fertilizer-holding capacity, thereby indirectly promoting crop growth [38]. Similarly, carbon-based microscale materials, such as micronized carbon black, improve soil porosity and aeration, creating an optimal environment for root development in crops like rice. At the nanoscale, carbon nanomaterials, such as carbon nanotubes and graphene, possess extraordinary physicochemical properties that benefit rice cultivation. Carbon nanotubes have been shown to enhance root water absorption significantly, particularly under drought conditions, thereby improving rice drought tolerance [39,40]. Graphene, by interacting with soil organic matter, increases the bioavailability of trace nutrients to rice plants, further improving their growth and productivity [41,42].

#### 2.1.3. Composite and Functionalized Materials

Composite and functionalized materials, both at micro-nanoscale levels, have gained prominence in rice agriculture due to their superior performance in soil remediation, plant protection, and controlled nutrient release. In rice production, composite materials with controlled-release properties have emerged as key technologies for improving fertilizer utilization efficiency and promoting sustainable agricultural practices. For example, biodegradable dextrin-based microgel composites allow for the dual slow release of fertilizers, extending nutrient availability and minimizing nutrient loss. This enhances rice growth and provides a sustainable fertilizer supply for agriculture [43]. Similarly, a slow-release nitrogen fertilizer system based on carboxymethyl cellulose (CMC) grafted with polyacrylamide (PAM) regulates nitrogen release through water absorption and swelling, improving nitrogen use efficiency in rice and reducing nitrogen loss to the environment [44]. Micro–nanoscale materials also perform exceptionally well in urea-controlled release systems, enabling prolonged nutrient release and reducing fertilizer waste. For instance, urea-loaded hydrogels fabricated using 3D printing techniques, such as agar-urea hydrogels, enable precise nitrogen release, thereby enhancing nitrogen uptake, crop resilience, and growth efficiency in rice [45]. These studies highlight the significant contributions of composite materials to increasing fertilizer efficiency, improving soil quality, and promoting sustainable crop production. Moreover, the multifunctionality of composite materials extends beyond nutrient delivery. They also serve as environmentally friendly pesticide carriers, supporting both yield enhancement and quality improvement in rice cultivation. Therefore, the application of composite micro–nanomaterials in rice agriculture demonstrates their immense potential in improving crop growth, enhancing stress tolerance, and advancing soil health. As such, these materials represent a critical innovation for sustainable agricultural development.

### 2.2. Functions of Micro–Nanomaterials in Agriculture

#### 2.2.1. Enhanced Fertilizers and Nano-Pesticides

Micro–nanomaterials play a crucial role in the development of enhanced fertilizers and pesticides. For example, micronized rice bran has been shown to improve its physicochemical and functional properties, enhancing its nutritional value and utility in promoting rice growth [46]. Micronization not only reduces particle size, but also increases water solubility and nutrient release, benefiting rice growth. Nanoscale zero-valent iron (nZVI) is another promising agricultural material that has been proven to significantly improve rice yield and nutrient content. Studies indicate that rice seeds treated with nZVI exhibit higher biomass and improved leaf morphology, primarily due to enhanced photosynthetic efficiency [19]. Furthermore, nZVI promotes the accumulation of macro-micronutrients in the soil, enhancing the overall nutritional quality of rice [23]. Nano-pesticides improve dispersibility and targeting, enabling effective pest and disease control while reducing the quantity of chemicals required. In comparison, micro-pesticides are well-suited for surface application in field crops. Their larger particle size minimizes drift and enhances adherence to leaf surfaces, making them effective for broad coverage against pests and diseases [47]. An innovative example is the micro–nanoscale bubble aeration irrigation technology, which generates nanoscale or micro–nanoscale bubbles to prolong dissolved air retention in water. This improves the growing environment for rice, thereby increasing yield. In practice, Xiao et al. demonstrated that coupling micro–nanoscale bubble devices with nitrogen application enhanced nitrogen use efficiency and provided new insights into the application of this technology [24].

#### 2.2.2. Enhanced Stress Tolerance

Micro–nanomaterials are particularly effective in enhancing rice resilience to various stresses. According to Wang et al., engineered nanomaterials improve water and nutrient absorption by rice roots and strengthen the plant’s antioxidant system, aiding growth under drought, salinity, and heavy metal stress [25]. For example, selenium nanomaterials effectively improve rice disease resistance and nutrient absorption, enhancing resistance to rice blast while improving nutritional quality, benefiting human health [26]. Similarly, manganese ferrite nanoparticles show potential in improving drought tolerance by enhancing water retention and activating the antioxidant enzyme system, helping rice maintain growth in arid conditions [48]. Silicon dioxide nanoparticles increase rice resistance to pests like the brown planthopper by altering the physical structure of the plant surface [49]. Riseh et al. also highlighted the role of micro-/nano-encapsulation technologies in slowly releasing biocontrol agents, enhancing rice immunity against biotic stressors [50]. Additionally, micro–nanomaterials can alleviate the effects of heavy metal pollution on rice. Firstly, the protective effect on the root system, research shows that TiO_2_ nanoparticles can form a protective film on the root surface and reduce the inward flow of Cd^2+^ [51], and SiO_2_ nanoparticles can reduce the amount of Cd accumulation in the apical meristematic zone [52]. Secondly, to regulate the secretion of root secretion, nanomaterials promote the synthesis and secretion of organic acids by activating plant root metabolic pathways, and organic acids form stable metal–ligand complexes with heavy metal ions in the soil, reducing the bioeffectiveness and toxicity of heavy metals [53,54]. In addition, some micro–nanomaterials can adsorb and passivate heavy metals. For instance, biochar-based phosphorus-rich nanomaterials reduce heavy metal pollution and improve the structure of soil microbial communities, thereby improving the growth quality and resistance of rice. Biochar, with its highly porous structure and huge specific surface area, is able to capture heavy metal ions by physisorption and chemisorption. In addition, phosphorus groups in phosphorus-rich biochar can form stable chemical bonds with heavy metal ions (e.g., lead, cadmium, mercury, etc.) to further immobilize these harmful ions. Elemental phosphorus can also react with heavy metal ions to produce insoluble phosphate precipitates, thereby significantly reducing the bioavailability and mobility of heavy metals. This dual action not only reduces the toxicity of heavy metals to rice, but also improves the soil environment, promotes the diversity and activity of the soil microbial community, and ultimately improves the quality of rice growth and resistance to adversity [55]. In summary, micro–nanomaterials show great promise in improving rice resilience against pests, drought, and heavy metal pollution while enhancing nutritional quality. These advancements have far-reaching implications for agricultural productivity and food security.

#### 2.2.3. Seed Germination and Photosynthesis Regulation

Nanomaterials have demonstrated significant advantages in promoting rice seed germination, storage resistance, and seedling development. Copper sulfide nano-dispersions, for example, reduce seed decay rates and enhance germination vigor and growth rate [56]. Zinc carbonate nanoparticles suppress fungal pathogens on seed surfaces and in growing environments while serving as a zinc source, improving seedling growth and zinc nutrition [57]. Chitosan nanoparticles (ChNP) have also been used in seed treatment, with studies showing that optimal concentrations significantly enhance germination rates and seedling growth, even under adverse conditions [27]. Nano-coatings for seeds integrate insecticides, fungicides, and fertilizers, providing enhanced stress tolerance and precise nutrient delivery. For instance, a metal–organic framework (MOF) called UIO-66, loaded with the fungicide Imazalil and protected by a tannic Acid-Zn coating, allows for pH- and temperature-triggered release, inhibiting Fusarium proliferatum spore growth [58]. Biodegradable coatings combining nano-clay, hydrogels, and starch enable slow-release zinc delivery, improving germination rates in rice [59]. Nanomaterials also influence photosynthesis and overall growth. For instance, the foliar application of nano-calcium oxide fertilizers optimizes nutrient absorption and metabolic processes, significantly increasing rice yield [28]. Nano-iron fertilizers boost chlorophyll content and biomass accumulation in seedlings, enhancing photosynthetic performance [35].

Microscale materials have also gained attention in seed treatments. The application of micron-sized silicate particles and carbon particles in seed coating can significantly enhance seed germination rates and early growth performance [60,61,62]. Firstly, silicate particles and carbon particles are capable of slowly releasing nutrients while adsorbing harmful ions in the soil, thereby providing a favorable environment for seed germination and seedling growth. These harmful ions primarily include heavy metal cations (e.g., Cd^2+^, Pb^2+^, As^3+^) and metalloid pollutants. Silicate particles capture heavy metal ions through ion exchange, and the ^-^OH groups within their layered structures can form stable complexes with metal ions, thereby reducing the bioavailability of heavy metals [63]. Carbon particles, on the other hand, further immobilize harmful ions through π-π interactions and surface functional groups (e.g., carboxyl and hydroxyl groups) via physical adsorption and chemical chelation [64]. Furthermore, micro-materials improve photosynthesis by reflecting sunlight, increasing light intensity and biomass accumulation under suitable conditions.

#### 2.2.4. Nano-Sensors in Precision Agriculture

Nano-sensors have emerged as cutting-edge tools for the real-time monitoring of soil, water, and plant health. These sensors can detect soil moisture, heavy metals, organic pollutants, nutrient levels, and environmental conditions, providing precise data to optimize soil management, irrigation, pest control, and climate adaptation. For example, Dwi Novianto et al. [62] developed soil moisture sensors that regulate water levels based on rice root growth characteristics, minimizing water waste and enabling precision irrigation. To combat rice kernel smut, Kritika Rana’s team [29] developed a graphene-based electrochemical DNA biosensor that rapidly and accurately detects pathogens, reducing detection time and cost compared to conventional methods. In heavy metal pollution monitoring, Tedrick Thomas et al. [30] designed a plant-based nano-biosensor that utilizes the natural arsenic absorption capacity of wild plants. This non-invasive sensor tracks arsenic dynamics in plant roots, providing insights into the effects of arsenic pollution on rice growth and informing remediation strategies. In summary, nano-sensors tailored for rice cultivation support precision agriculture by improving soil quality, yield, and crop quality, offering transformative solutions for sustainable farming.

## 3. Potential Risks of Micro–Nanomaterials on Rice Growth

Despite the immense potential of micro–nanomaterials in enhancing rice resilience, improving soil quality, and promoting food security, their application in agriculture is accompanied by significant environmental and ecological risks. This section explores these risks from three critical perspectives.

### 3.1. Toxic Effects on Rice Growth

**Figure 1:**
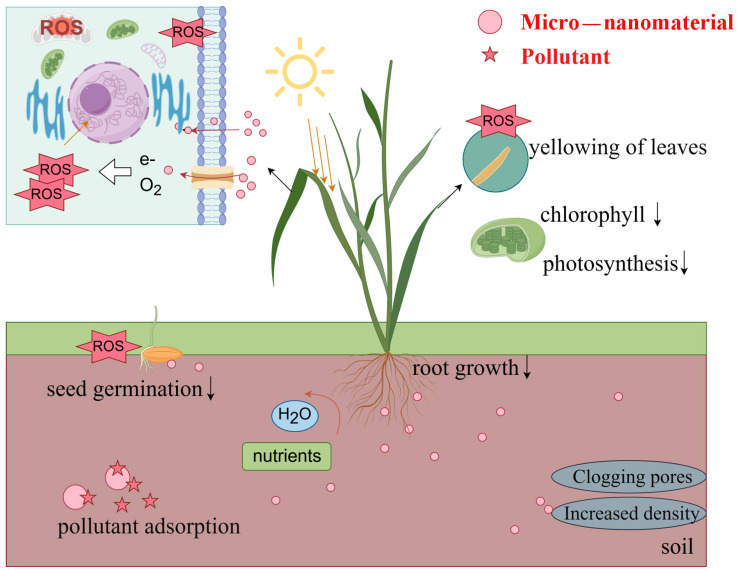
Toxic effects of micro–nanomaterial on rice, created using Figdraw.

The accumulation of micro–nanomaterials in rice plants can trigger various toxic reactions, particularly at high concentrations (Figure 1). Many nanomaterials, such as metal oxides and carbon nanotubes, exhibit high chemical reactivity, promoting the generation of reactive oxygen species (ROS). Excessive ROS levels can induce oxidative stress, damaging cellular structures, including membranes, proteins, and DNA, ultimately inhibiting plant growth and development. Qian et al. reported that the accumulation of certain nanomaterials in soil directly impairs root development in rice, limiting the absorption of water and nutrients [65]. Similarly, Wang et al. [66] observed that exposure to specific nanomaterials, such as zinc oxide and silver nanoparticles, led to typical toxic effects in rice, including growth inhibition and leaf chlorosis. Such damage to cellular structures and metabolic processes directly reduces rice yield and stress resistance. Chen et al. highlighted the adverse impact of microplastics in paddy soils, demonstrating that they inhibit rice growth, restrict root elongation, and reduce both yield and grain quality [67]. Additionally, microplastics alter soil physicochemical properties, decreasing soil fertility and microbial activity, further exacerbating growth suppression. Yang found that polyethylene microplastics significantly hinder rice seed germination, reduce biomass accumulation [68], and degrade rhizosphere conditions by limiting soil aeration and nutrient availability. Ma et al. further investigated the metabolic disruptions caused by microplastics in rice. They observed reduced chlorophyll content and inhibited photosynthesis, which negatively affected rice growth and yield. Furthermore, microplastics were shown to interfere with nutrient uptake, exacerbating deficiencies and growth limitations. These findings highlight the urgent need to evaluate the ecological risks of microplastics and their impacts on rice productivity [69].

### 3.2. Accumulation in Soil and Water and Long-Term Ecosystem Impacts

The use of nanomaterials in agriculture, particularly for irrigation and soil remediation, may lead to their accumulation in soil and water. Usman et al. discussed the potential ecological consequences of nanomaterials in agricultural systems, noting that materials like titanium dioxide and silica nanoparticles persist in soil for extended periods. These materials can enter rice plants through root uptake and gradually accumulate within the plant system. While short-term benefits may be observed, prolonged accumulation can disrupt soil microbial communities, alter soil physicochemical properties, and compromise ecological balance [70]. Zhou et al. pointed out that nanomaterials entering water bodies not only affect rice, but may also propagate through the food chain, posing risks to aquatic organisms such as fish and microorganisms [71]. The continued accumulation of nanomaterials could harm non-target species, including insects and birds, potentially threatening the health of entire ecosystems. These findings suggest that the long-term ecological impacts of nanomaterials on soil and water systems require greater attention. The unintended effects on soil quality, microbial dynamics, and food webs underscore the need for comprehensive environmental monitoring and risk assessment.

### 3.3. Synergistic Pollution from Microplastics and Heavy Metals

Microplastic and heavy metal pollution are two interconnected environmental challenges in rice cultivation. Singh and Gurjar [72] emphasized that microplastics primarily enter agricultural soils through irrigation water, fertilizers, and pesticides, ultimately accumulating in crops. In addition to their direct toxic effects on rice, microplastics alter soil microbial communities and disrupt ecological functions, indirectly affecting rice growth. Heavy metals such as cadmium, lead, and arsenic also pose significant toxicity risks in paddy soils. Wahab et al. [73] highlighted that the combined presence of microplastics and heavy metals exacerbates environmental stress on rice, leading to more severe growth inhibition. This synergistic pollution amplifies physiological damage, increases toxicity accumulation in rice, and poses heightened risks to human health through the food chain. Dong et al. [74] investigated the co-pollution effects of microplastics and arsenic on rice and found that their combined presence aggravated root damage, suppressed photosynthesis, and impaired overall plant health. Microplastics not only directly affect rice growth, but also interact with heavy metals and other contaminants, leading to more pronounced toxic effects. Such findings underscore the need for integrated pollution management strategies to mitigate the compounded impacts of microplastics and heavy metals in rice production systems.

## 4. Global Research Trends and Hotspot Areas

This study analyzed 507 fully qualified articles retrieved from the Web of Science Core Collection (WoSCC) database using the following search strategy: ((TI = (rice or Oryza sativa)) AND TS = (plant)) AND TS= (nano or nanomaterial or nanoparticle or nanopparticle or micromaterial* or microplastic*)). The retrieved articles were subjected to bibliometric analysis using CiteSpace and VOSviewer.

### 4.1. Global Trends in the Impact of Micro–Nanomaterials on Rice

**Figure 2:**
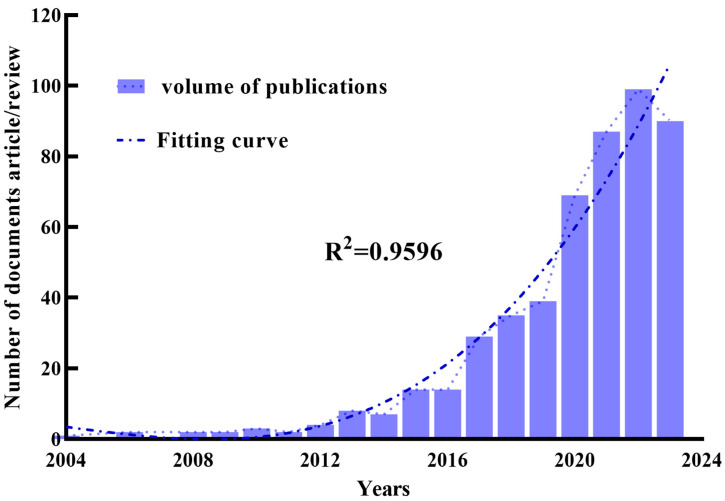
Annual publication volume and trends. Purple bars represent the number of papers related to micro–nanomaterials and rice per year. The blue dotted line represents the trend-fitted curve and the correlation coefficients (R^2^) is displayed in the figure.

In recent years, the integration of micro–nanomaterials into rice research has emerged as a prominent academic focus. Over the past 20 years, the volume of publications in this field has demonstrated a distinct upward trend, which can be categorized into three key phases: The Initiation Phase (2004–2015), the Acceleration Phase (2016–2017), and the Rapid Growth Phase (2018–2023) (Figure 2). During the Initiation Phase (prior to 2015), the annual growth in publications was relatively modest. Although researchers began to identify the potential environmental impacts of micro–nanomaterials, their specific interactions with rice had not been comprehensively investigated. Beginning in 2016, with the broader application of micro–nanotechnology, research efforts increasingly focused on evaluating the capacity of these materials to enhance rice quality [75,76], while simultaneously exploring their potential toxicological effects [77,78]. This period marked the onset of the Acceleration Phase, characterized by a significant increase in publication output. The Rapid Growth Phase (2018–2023) has been marked by an exponential rise in research activity and annual citations. The strong correlation between the number of publications and citation frequency during this phase underscores the growing recognition and academic impact of research in this domain. These trends reflect the expanding focus of the scientific community on both the opportunities and challenges presented by micro–nanomaterials in rice cultivation.

**Figure 3:**
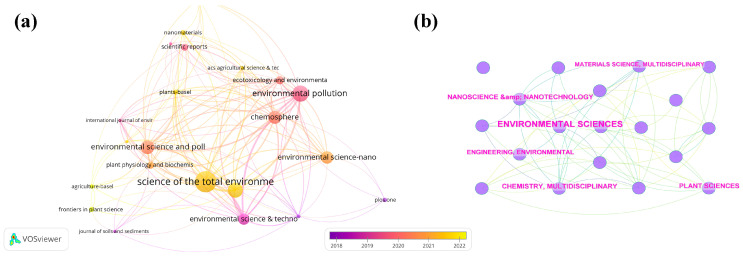
(**a**) Main journals clustered using VOSviewer. (**b**) Subject categories with more than 20 publications using CiteSpace.

To date, over 150 journals in total have published articles in this specialized field. Figure 3a shows the journals with more than five publications in the field, with Science of the Total Environment leading the list (*n* = 31), followed by the Journal of Hazardous Materials (*n* = 25), and Environmental Pollution (*n* = 22). Among these, the Journal of Hazardous Materials stands out with the highest impact factor (IF) of 12.2 and a h-index of 235, underscoring its prominence in the field.

Table 2 presents the ten most cited journals, showing that Environmental Science & Technology is the most frequently cited, with a total of 276 citations. Figure 3b further identifies the disciplines contributing significantly to this research domain, specifically those with more than 20 published articles. The top five disciplines are Environmental Science (*n* = 218), Nanoscience and Nanotechnology (*n* = 59), Environmental Engineering (*n* = 52), Plant Science (*n* = 51), and Multidisciplinary Chemistry (*n* = 48).

This analysis of journal performance provides valuable insights for researchers aiming to select appropriate venues for their submissions. Notably, the journals with the highest citation counts listed in Table 2 closely align with those identified in Figure 3a, further highlighting the concentrated research focus within this field. This alignment reflects both the interdisciplinary nature of the topic and the consistent influence of leading journals in driving advancements in this area.

Within this research field, a total of 2075 authors have contributed relevant studies, with Xing Baoshan from the University of Massachusetts being the most prolific (n = 17), followed by Li Bin from Zhejiang University (n = 14) and Rui Yukui from China Agricultural University (n = 14). Xing Baoshan’s work has primarily focused on elucidating the toxicity mechanisms of heavy metals and nanomaterials in rice [79,80,81,82], investigating their effects on rice growth and potential applications in mitigating environmental stress. His team has also explored innovative uses of nanomaterials, such as the development of slow-release fertilizers designed to alleviate arsenic toxicity in rice [83,84]. Li Bin has concentrated on the agricultural applications of biosynthesized nanomaterials, with particular emphasis on developing environmentally friendly nanomaterials to suppress rice pathogens [85,86,87,88] and enhance drought tolerance [89]. Despite these advancements, significant challenges remain, especially in scaling these technologies for practical agricultural applications. Similarly, Rui Yukui has made notable contributions to understanding the interactions between nanomaterials and rice physiology. His research has examined the effects of nanoparticles on nutrient uptake, stress resistance, and soil–rice interactions, providing valuable insights into how these materials influence plant performance under various environmental conditions.

The findings from these studies underscore both the immense potential and the inherent challenges of applying micro–nanomaterials in rice cultivation. For instance, while their applications show promise, there remains a critical gap in the systematic understanding of how these materials affect soil microbial communities and their interactions with plant physiological processes. This knowledge gap highlights the urgent need for further research to unravel the comprehensive impacts of micro–nanomaterials on plants and soil ecosystems. Addressing these gaps would not only advance our fundamental scientific understanding, but also pave the way for the development of sustainable and effective agricultural technologies.

### 4.2. Research Hotspots and Temporal Trends

This study systematically analyzed keywords and research clustering to identify the current research hotspots, evolutionary trends, and future directions in the study of micro–nanomaterials in rice cultivation [90,91].

#### 4.2.1. Key Research Themes

**Figure 4:**
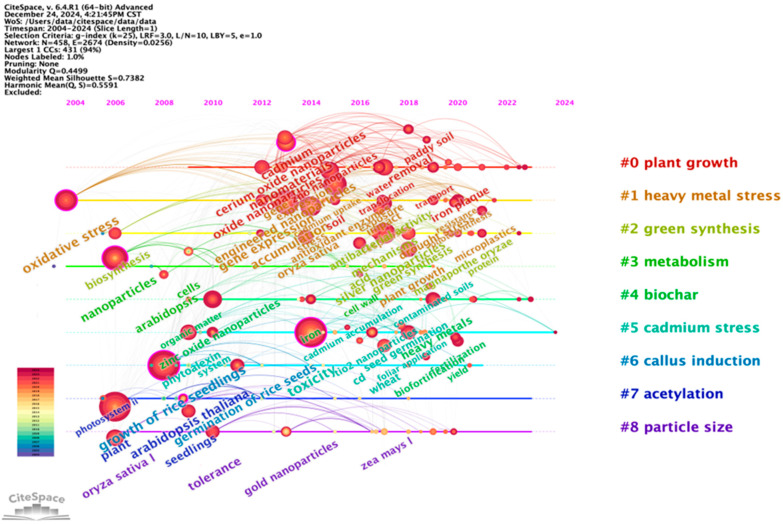
Co-occurring keywords over time, clustered using CiteSpace.

As illustrated in Figure 4, the field revolves around several core themes, including plant toxicity [92,93,94], plant absorption [95,96,97], and the effects of cadmium stress [98,99,100]. These topics reflect the growing academic interest in understanding the ecological and physiological impacts of micro–nanomaterials on rice.

The timeline visualization of research clusters (Figure 4) reveals the chronological development and evolution of specific topics. For instance, the research theme of “Chilling stress” emerged after 2012, reaching its peak in 2021. Recent studies have focused on the potential of nanomaterials to enhance rice survival under low-temperature stress, highlighting this as a critical area of application aimed at improving rice resilience in ad-verse environmental conditions [101]. Another significant research focus is on the “early growth of rice”. Initial studies in this area investigated the oxidative responses of rice exposed to nanomaterials during its early growth stages. By 2023, emphasis had shifted toward exploring how nanotechnology could enhance stress tolerance during early development. For example, certain nanoparticles have demonstrated the ability to mitigate cadmium toxicity in rice seedlings, offering promising solutions for cultivation in contaminated environments [102]. The study of photosynthesis in rice represents another pivotal research theme, particularly prominent before 2015. Given the central role of chloroplasts in photosynthesis, their health and efficiency are critical for plant growth and metabolism. Research has shown that small quantities of carbon nanotubes can improve the photosynthetic efficiency of rice chloroplasts, thereby boosting overall plant performance [103].

A particularly noteworthy cluster is “green synthesis”. This cluster reflects a growing interest in the development of environmentally friendly micro–nanomaterials, driven by concerns about minimizing their ecological risks during both production and application. Green synthesis approaches aim to leverage sustainable methodologies to enhance the positive impacts of these materials on plant growth while simultaneously mitigating their environmental footprint (Table 3). This shift underscores the increasing emphasis on balancing the benefits of nanomaterial applications in agriculture with the need for sustainability and ecological safety.

#### 4.2.2. Evolution of Research Through Co-Citation Analysis

**Figure 5:**
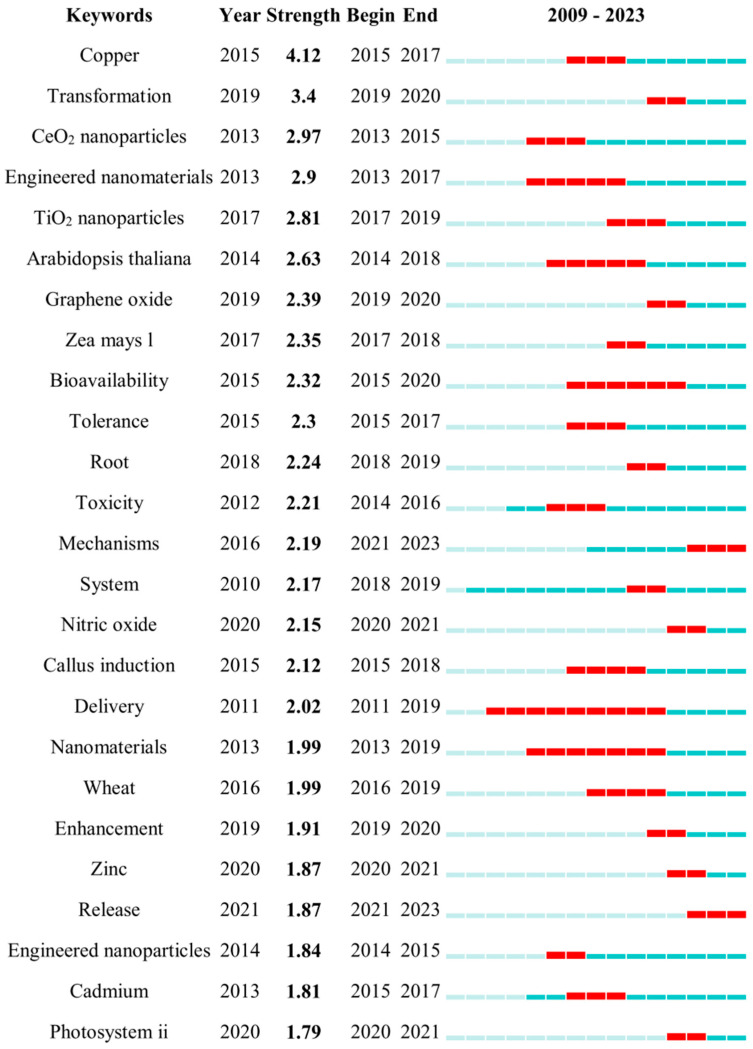
Top 25 keywords with the strongest citation bursts by CiteSpace. A blue line indicates the timeline, and the bars in red stand for a burst period that includes the beginning year, the end year, and the burst duration of the keywords.

The co-citation timeline view generated using CiteSpace (Figure 5) offers a comprehensive overview of the evolution of the research in this field.

Early research: Initial studies primarily focused on the development and optimization of micro–nanomaterials, characterizing their physicochemical and biological properties, and evaluating their preliminary applications in rice disease management. For instance, Bin Li and colleagues developed micro–nanomaterials to enhance the specificity and bioactivity of pesticides, thereby reducing environmental pollution [85,86,87,88,89]. Concurrently, Baoshan Xing’s research team applied micro–nanomaterials in rice growth environments, systematically investigating their effects on rice development and exploring their potential to improve crop resilience under environmental stress [79,80,81,82,83,84]. Building on this foundation, Lin et al. expanded the scope by examining the potential of nanomaterials to enhance nutrient uptake and water-use efficiency in rice [95,96,97]. 

Current research: Research hotspots have gradually shifted toward addressing issues such as heavy metals, nanoplastics, and biochar, reflecting evolving priorities in the field.

Heavy metal stress: Among various heavy metals, cadmium has attracted significant attention due to its extreme toxicity, its capacity to damage DNA, and its disruption of enzymatic activities [116,117]. Industrial processes have led to increased cadmium contamination in soils, and its high mobility amplifies the risks of bioaccumulation along the food chain [118,119,120]. Research has increasingly focused on understanding cadmium’s impacts on rice and mitigating its effects through innovative micro–nanomaterials.

Nanoplastics: Nanoplastics, primarily derived from the degradation of microplastics, have emerged as a critical area of study. Unlike microplastics, nanoplastics’ smaller size allows them to infiltrate biological systems more easily. Their high surface area and porosity enable them to adsorb toxic substances and biomolecules, posing significant ecological risks [121,122]. These unique properties have prompted investigations into their behavior, toxicity, and long-term effects on soil and plant systems.

Biochar: Biochar, a nanomaterial characterized by its high surface area, porous structure, and abundance of functional groups, has demonstrated significant potential for enhancing soil properties and plant performance. Research has shown its effectiveness in improving soil structure, increasing fertility, remediating heavy metal and pesticide residues, and enhancing stress tolerance in plants. Recent studies have focused on modifying biochar to optimize its performance [123] and developing biochar-based fertilizers aimed at improving rice resilience under various stress conditions [124].

Emerging themes: One of the latest themes in this field involves luminescent micro–nanomaterials, which have garnered attention for their unique afterglow properties. These materials emit red and blue afterglow spectra that overlap with chlorophyll’s ab-sorption spectrum, effectively extending the photoperiod for rice. Preliminary findings suggest that luminescent nanomaterials could enhance photosynthesis and improve rice growth efficiency. However, concerns remain about the potential adverse effects of pro-longed light exposure, which may disrupt dark-phase metabolism and interfere with the synthesis and accumulation of key nutrients in plants. Further research is necessary to evaluate the long-term implications of these materials on rice growth and productivity.

#### 4.2.3. Future Research Directions

The field of micro–nanomaterials in rice research has evolved from an initial focus on material synthesis and characterization to a broader multidisciplinary approach emphasizing functional optimization and environmental safety. Moving forward, future research is expected to prioritize several key areas. Comprehensive studies are needed to systematically assess the ecological effects of micro–nanomaterials, particularly their interactions with soil microbial communities, plant physiology, and ecosystem dynamics. Such research would provide critical insights into the broader implications of these mate-rials for agricultural systems. At the same time, sustainable applications in agriculture should be prioritized, with a focus on eco-friendly and green synthesis methods to reduce environmental risks while enhancing material functionality. The development of such approaches aligns with the growing demand for sustainability and environmental stewardship in modern agriculture.

Additionally, integrating nanotechnology with other scientific fields, such as bioengineering, environmental science, and synthetic biology, holds significant promise for unlocking novel agricultural applications. For example, combining nanotechnology with advanced gene-editing tools or soil science could lead to innovative strategies for improving crop resilience, nutrient use efficiency, and stress tolerance. Heavy metal remediation remains a particularly urgent area of focus, with an emphasis on leveraging nanotechnology and biochar to mitigate the effects of toxic metals like cadmium. The modification of biochar using nanomaterials to enhance its adsorption and remediation capabilities offers a promising direction for reducing heavy metal uptake in rice and addressing soil contamination issues.

Finally, the safety and risk assessment of emerging materials, such as nanoplastics and luminescent nanoparticles, represents a critical research priority. Comprehensive evaluations of their long-term ecological impacts, persistence in soil and water systems, bioaccumulation potential, and potential risks to plant and human health are essential for ensuring the safe application of these materials in agriculture. Addressing these challenges will be fundamental to advancing the field and fostering the development of innovative and sustainable technologies while safeguarding environmental and human health.

## 5. Current Technical Bottlenecks

Despite their significant potential in improving crop yield, nutrient efficiency, and stress tolerance, the application of micro–nanomaterials in agriculture, particularly rice farming, faces several key technical challenges. These challenges primarily stem from gaps in toxicity and ecological assessment methods, high production costs, and the complexities of the interactions within agricultural ecosystems.

### 5.1. Insufficient Methods for Toxicity and Ecological Risk Assessment

One of the critical challenges is the lack of standardized frameworks for assessing the ecological toxicity and environmental impacts of micro–nanomaterials. Due to the highly variable behavior of these materials under different concentrations, environmental conditions, and application contexts, traditional toxicity assessment methods fail to capture their complexity adequately.

Research indicates that the absence of precise assessment methods limits the broader agricultural adoption of micro–nanomaterials and poses significant challenges for evaluating their long-term ecological safety. For crops like rice, the ecological effects of nanomaterials can vary significantly depending on soil types and climatic conditions. This variability highlights the need for more sophisticated tools to evaluate the potential risks and long-term impacts of these materials [125,126,127].

Without robust evaluation frameworks, the potential hazards posed by micro–nanomaterials, such as oxidative stress, soil microbial imbalances, and unintended environmental consequences, remain poorly understood. Consequently, future research must prioritize the development of precise context-sensitive toxicity assessment methods to ensure the safe and effective use of these materials in agriculture.

### 5.2. High Production Costs

The high production costs of micro–nanomaterials remain a significant barrier to their widespread application in rice farming. The manufacturing of these materials often requires sophisticated processes and expensive equipment, resulting in production costs that far exceed those of traditional fertilizers and pesticides [128].

In the early stages of production, low output volumes lead to high unit costs, creating significant economic pressure for agricultural applications of nanotechnology. Furthermore, scaling up production while maintaining strict control over particle size, morphology, and distribution poses additional technical and economic challenges [129].

The initial investment required for applying nanotechnology in agriculture also includes the costs of specialized equipment and technical training for personnel. These high upfront expenses are particularly burdensome for resource-limited farmers, who may lack the financial means to adopt such technologies [130].

Additionally, while nanomaterials have demonstrated potential for improving crop yields and fertilizer efficiency, the economic return on investment often requires years to materialize. This extended recovery period may deter farmers, who typically rely on quick financial returns to sustain their operations [131].

Finally, the uncertain environmental impacts of nanomaterials, particularly their long-term effects on soil microbial communities and ecosystems, could lead to additional costs for remediation and regulatory compliance. Thus, reducing production costs and mitigating the financial risks associated with nanomaterial adoption are crucial for their broader acceptance in agriculture.

### 5.3. Complex Interactions Within Agricultural Ecosystems

The long-term interactions of micro–nanomaterials with soil, water, and plants remain poorly understood, presenting a significant challenge to their agricultural application.

Once introduced into the soil, micro–nanomaterials interact with soil particles, microbes, and plant roots, influencing the soil’s physicochemical properties, microbial community structures, and crop growth. However, research on these interactions is still limited, particularly regarding the cumulative and long-term ecological effects [128].

Similarly, when micro–nanomaterials enter water bodies, they may disperse into surrounding ecosystems, potentially impacting aquatic organisms. For example, silver nanoparticles and copper oxide nanoparticles have been shown to affect the growth and metabolism of rice. However, the lack of long-term ecological tracking studies makes it difficult to evaluate their persistence, degradation, and potential toxicity in water systems [132,133].

Furthermore, the impacts of nanomaterials on rice are not limited to their direct interactions with plants. These materials can participate in complex transfer processes within the plant–soil-water continuum. For instance, copper oxide and silver nanoparticles have been found to accumulate in rice roots and translocate within the plant via bioconcentration, potentially affecting growth and metabolism [133]. However, the long-term implications of such accumulation, particularly their effects on rice productivity and their transfer through the food chain to human consumers, remain unclear.

Current studies predominantly focus on short-term experiments, with insufficient attention paid to the cumulative effects of micro–nanomaterials over extended periods. Comprehensive long-term studies are essential to evaluate their ecological and environmental impacts more thoroughly.

## 6. Conclusions

The application of micro–nanomaterials in rice cultivation represents a growing interdisciplinary frontier, integrating agriculture, materials science, environmental science, and nanotechnology. These materials show great potential to accelerate agricultural modernization, particularly in Asia, by improving rice growth, stress tolerance, nutrient ab-sorption, and soil health.

Nanomaterials enhance rice productivity through mechanisms such as increased fertilizer efficiency, reduced environmental pollution, optimized metabolic processes, and improved photosynthesis. They also stimulate root development, enhance microbial activity in the rhizosphere, and promote healthier soil ecosystems. Moreover, their role in soil improvement and environmental remediation—such as addressing saline-alkali and heavy metal-contaminated soils—is particularly noteworthy. By modifying soil structure and adsorbing contaminants, these materials support sustainable agricultural practices.

However, long-term ecological risks, such as the potential effects on soil microbial com-munities, biodiversity, and soil health, remain poorly understood. Future research should prioritize evaluating these risks, understanding their impact on carbon cycling and nutrient dynamics, and developing standardized safety assessment methods to enable large-scale applications.

The integration of nanotechnology with smart agriculture technologies, such as IoT, AI, and big data, offers exciting opportunities for innovation. IoT-enabled nanosensors, for instance, can monitor soil conditions in real-time, optimize irrigation and fertilization, and diagnose pests and diseases, promoting precision agriculture and reducing environ-mental impacts.

To fully realize the potential of micro–nanomaterials, future efforts must focus on designing multifunctional eco-friendly materials with superior nutrient delivery and stress resistance while minimizing environmental risks through biodegradable properties. Advances in green chemistry and nanomaterial science will be essential for developing cost-effective and scalable manufacturing techniques and for creating sustainable agricultural solutions. Addressing the current challenges, such as high production costs and scalability, will be critical for the widespread adoption of these technologies, paving the way for modernized and sustainable rice cultivation.

## Figures and Tables

**Table 1:** Functions of micro–nanomaterials in agriculture.

Category	Functions	Detailed Insights	Examples	References
Enhanced Fertilizers and Nano-Pesticides	(a) Improve dispersibility and targeting of fertilizers and pesticides.	Nano-fertilizers reduce nutrient loss and improve efficiency through controlled-release mechanisms, addressing overuse of fertilizers and environmental pollution.	Nano zero-valent iron (nZVI), nano-pesticides, micronized pesticides.	[23,24]
	(b) Increase nutrient utilization efficiency and reduce chemical usage.			
	(c) Provide long-term nutrient release for better yield and quality.			
Improved Stress Tolerance	(a) Enhance water and nutrient absorption.	Nanomaterials enhance resilience to stress by boosting antioxidant systems, immobilizing toxins in soil, and supporting microbial communities for improved plant health.	Selenium nanoparticles, silicon dioxide nanoparticles, manganese ferrite nanoparticles, nano-biochar.	[25,26]
	(b) Strengthen antioxidant systems to combat drought, salinity, and heavy metal stress.			
	(c) Boost resistance to pests while improving nutritional quality of crops.			
Seed Germination and Photosynthesis Regulation	(a) Improve seed germination rates and seedling growth.	Enhance germination and growth by improving nutrient delivery and pathogen resistance. Boost photosynthesis efficiency via foliar application of nanomaterials, increasing yield.	Copper sulfide nano-dispersions, zinc carbonate nanoparticles, chitosan nanoparticles, nano-calcium oxide.	[27,28]
	(b) Enhance seed storage resistance.			
	(c) Boost photosynthesis efficiency, promoting biomass accumulation.			
Nano-Sensors for Precision Agriculture	(a) Real-time monitoring of soil, water, and plant health.	Provide precise, real-time data for optimizing irrigation, pest control, and fertilization. Sensors enable the early detection of pathogens and soil issues to support sustainable agriculture.	Nano-moisture sensors, graphene-based electrochemical DNA sensors, plant-based nano-biosensors.	[29,30]
	(b) Detect heavy metals, pathogens, and nutrient levels.			
	(c) Optimize irrigation, fertilization, and pest control based on precise data.			

**Table 2:** Top 10 cited journals.

Top	Journal	Citations	IF 2024	JCR ^1^ 2024	H-Index ^2^
1	*Environmental Science & Technology*	276	10.8	Q1	345
2	*Environmental Pollution*	275	7.6	Q1	194
3	*Chemosphere*	266	8.1	Q1	212
4	*Science of The Total Environment*	257	8.2	Q1	205
5	*Journal of Hazardous Materials*	254	12.2	Q1	235
6	*Ecotoxicology and Environmental Safety*	219	6.2	Q1	110
7	*Plant Physiology and Biochemistry*	206	6.1	Q1	105
8	*Environmental Science and Pollution Research*	200	5.8	Q1	82
9	*Frontiers in Plant Science*	186	4.1	Q1	83
10	*Plant Physiology*	181	6.5	Q1	276

^1^ JCR (Journal Citation Reports) is a ranking system within the WOS database that categorizes journals in each discipline into Q1–Q4 levels, with Q1 representing the highest quality. ^2^ The h-index indicates that a journal has at least h articles cited at least h times.

**Table 3:** Green synthetic nanoparticles.

NO.	NPs	Synthetic Sources	Characteristic	Fields of Application	References
1	Au-NPs	Extracts of *Cucurbita pepo* L. Leaves		Nanocatalysis, biomedical	[104]
2	Au-NPs	Methanolic leaf extract of *Moringa oleifera*	Antibacterial, antioxidant, haemocytotoxic, lower cytotoxicity, favors nerve cell regeneration, good photocatalytic efficiency	Biomedical, therapeutic applications	[105]
3	Au-NPs	*Murraya koenegii* Spreng. Leaf Extract		Fabrication of new photonic devices, cancer diagnostics	[106]
4	Ag-NPs	Peel extracts of citrus macroptera fruit		Industrial applications	[107]
5	Ag-NPs Ag/AgFeO_2_	Teucrium polium plant	Antifungal, antibacterial	Biomedical	[108]
6	Ag–Curcumin Nanoconjugates	Curcumin	Biocompatible, antimicrobial activity		[109]
7	Synthesis of Hybrids from Lignocelluloses and Silver	Dewaxed cotton straw powder		Biomedical	[110]
8	ZnO-NPs	Paspalidium Flavidum	Antimicrobial activity, photocatalytic activity, biocompatibility		[111]
9	ZnO-NPs	*Artocarpus gomezianus*	Antioxidant, photocatalytic activity, luminescence		[112]
10	Se-NPs	Garlic cloves	Low cytotoxicity, eco-friendly	Agriculture	[113]
11	CaO-NPs	Leave extract of *Rhododendron arboreum*	Adsorption, antibacterial, catalytic		[114]
12	Pd-NPs	Papaya peel	Catalysis		[115]

## Data Availability

The data that support the findings of this study are available from the corresponding author upon reasonable request.

## Abbreviations

The following abbreviations are used in this manuscript:

NPs	Nanoparticles
WoSCC	Web of Science Core Collection
CMC	Carboxymethyl cellulose
PAM	Polyacrylamide
nZVI	Nanoscale zero-valent iron
ChNP	Chitosan nanoparticles
MOF	Metal–organic framework
ROS	Reactive oxygen species
